# Determination of the Potential Tumor-Suppressive Effects of *Gsdme* in a Chemically Induced and in a Genetically Modified Intestinal Cancer Mouse Model

**DOI:** 10.3390/cancers11081214

**Published:** 2019-08-20

**Authors:** Lieselot Croes, Erik Fransen, Marieke Hylebos, Kimberly Buys, Christophe Hermans, Glenn Broeckx, Marc Peeters, Patrick Pauwels, Ken Op de Beeck, Guy Van Camp

**Affiliations:** 1 Center of Medical Genetics, University of Antwerp, Prins Boudewijnlaan 43/6, Edegem, BE-2650 Antwerp, Belgium; 2 Center for Oncological Research, University of Antwerp, Universiteitsplein 1, Wilrijk, BE-2610 Antwerp, Belgium; 3 StatUa Center for Statistics, University of Antwerp, Prinsstraat 13, BE-2000 Antwerp, Belgium; 4 Department of Pathology, Antwerp University Hospital, Wilrijkstraat 10, Edegem, BE-2650 Antwerp, Belgium; 5 Department of Medical Oncology, Antwerp University Hospital, Wilrijkstraat 10, Edegem, BE-2650 Antwerp, Belgium

**Keywords:** Gsdme, Dfna5, knockout, mouse model, colorectal cancer, intestinal cancer, Apc, AOM, azoxymethane, inflammation

## Abstract

*Gasdermin E* (*GSDME*), also known as *deafness autosomal dominant 5* (*DFNA5*) and previously identified to be an inducer of regulated cell death, is frequently epigenetically inactivated in different cancer types, suggesting that *GSDME* is a tumor suppressor gene. In this study, we aimed to evaluate the tumor-suppressive effects of GSDME in two intestinal cancer mouse models. To mimic the silencing of *GSDME* by methylation as observed in human cancers, a *Gsdme* knockout (KO) mouse was developed. The effect of GSDME on tumorigenesis was studied both in a chemically induced and in a genetic intestinal cancer mouse model, as strong evidence shows that GSDME plays a role in human colorectal cancer and representative mouse models for intestinal cancer are available. Azoxymethane (AOM) was used to induce colorectal tumors in the chemically induced intestinal cancer model (*n* = 100). For the genetic intestinal cancer model, *Apc^1638N/+^* mice were used (*n* = 37). In both experiments, the number of mice bearing microscopic proliferative lesions, the number and type of lesions per mouse and the histopathological features of the adenocarcinomas were compared between *Gsdme* KO and wild type (WT) mice. Unfortunately, we found no major differences between *Gsdme* KO and WT mice, neither for the number of affected mice nor for the multiplicity of proliferative lesions in the mice. However, recent breakthroughs on gasdermin function indicate that GSDME is an executioner of necrotic cell death. Therefore, it is possible that GSDME may be important for creating an inflammatory microenvironment around the tumor. This is in line with the trend towards more severe inflammation in WT compared to *Gsdme* KO mice, that we observed in our study. We conclude that the effect of GSDME in tumor biology is probably more subtle than previously thought.

## 1. Introduction

*Gasdermin E (GSDME)*, also known as *deafness autosomal dominant 5* (*DFNA5*), was discovered in our lab as a gene responsible for a specific form of nonsyndromic, autosomal dominant hearing loss [1]. Previously, we demonstrated that GSDME has the capacity to induce regulated cell death [2,3,4].

Since its discovery in 1998, a number of studies on GSDME have been published, pointing towards a possible involvement in cancer [2,3,4,5,6,7,8,9,10,11,12,13,14,15,16,17]. Moreover, genomic methylation screens unveiled *GSDME* as a possible tumor suppressor gene [11,13,14]. Furthermore, epigenetic silencing through *GSDME* methylation was previously shown in primary gastric [14], breast [6,7,12], and colorectal cancer [5,9,13]. In addition, *GSDME* expression was significantly downregulated, both in colon cancer samples and in colorectal cancer cell lines [13]. Finally, in vitro studies showed an increase in cellular invasiveness, colony numbers, colony size, and cell growth in colorectal cancer cell lines after *GSDME* knock-down [13]. Forced expression of GSDME, on the other hand, decreased cell growth and colony forming ability. In conclusion, these data suggested that *GSDME* is a tumor suppressor gene, which is often epigenetically inactivated through DNA methylation in different types of cancer.

In this study, we aimed to determine the potential tumor-suppressive effects of *Gsdme* both in a chemically induced and in a genetically modified intestinal cancer mouse model, given the strong evidence that GSDME plays a role in human colorectal cancer [5,9,13] and good, representative mouse models for intestinal cancer are available [18,19,20,21,22,23,24,25,26]. To mimic the silencing of *GSDME* by methylation, as observed in human cancers, a *Gsdme* knockout (KO) mouse model was developed.

For the chemically induced intestinal cancer model, azoxymethane (AOM) was used. AOM is a chemical agent that can initiate cancer by alkylation of DNA, thereby facilitating base mismatch [19]. The AOM model recapitulates many of the histopathological features associated with the multistage progression of human sporadic colorectal cancers [19,27]. Moreover, it has already been successfully used in numerous studies investigating factors that play a role in the modulation of tumor initiation and progression [28,29,30]. The model that was used in this study, with repeated intraperitoneal (i.p.) injections, is especially useful for studying factors that drive spontaneous tumor progression [19]. For the genetic intestinal cancer model, *Apc^1638N/+^* mice were used. Mutations in the *APC* gene are found in the earliest stages of the adenoma-carcinoma pathway and therefore play a crucial role in tumor formation and progression. The *Apc^1638N/+^* mouse model was chosen because it is a well-documented strain of genetically engineered *Apc* mice with a C57BL/6 background [20,21]. Compared to the frequently used *Apc^Min/+^* mice, *Apc^1638N/+^* mice have an attenuated intestinal phenotype with fewer tumors, occurring at a later time, which can progress into adenocarcinomas [20,21]. Therefore, *Apc^1638N/+^* mice are suitable for determining the effects of additional factors, such as *Gsdme*, in carcinogenesis. *Apc^1638N/+^* mice are known to progressively develop aberrant crypt foci, colonic polyps, and tumors of the small intestine, both benign adenomas and malignant adenocarcinomas, in the duodenum and jejunum [21,31]. In this study, we compared the number of mice bearing microscopic proliferative lesions, the number and type of lesions per mouse and the histopathological features of the adenocarcinomas between *Gsdme* KO and wild type (WT) mice.

## 2. Results

### 2.1. Validation of the Gsdme KO Mouse Model

**Figure 1:**
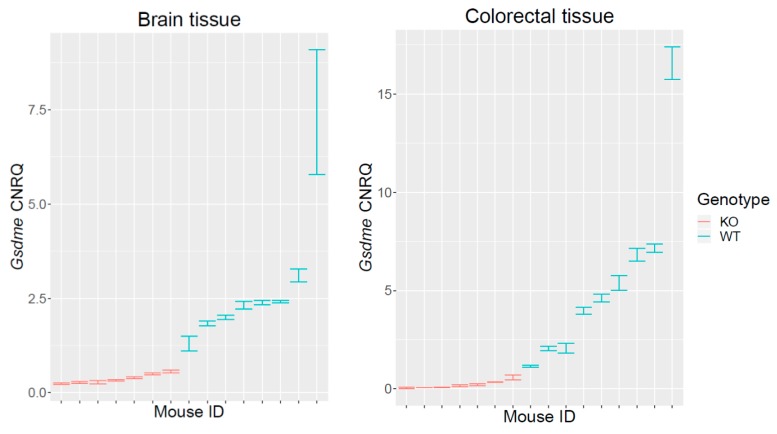
qRT-PCR analyses on *Gsdme* KO and WT mice. qRT-PCR analyses for *Gsdme* mRNA expression on *Gsdme* KO (*n* = 7) and WT (*n* = 9) mice, both on brain (*n* = 15) and colorectal (*n* = 16) tissues were performed. The Calibrated Normalized Relative Quantity (CNRQ) ± standard error (se) is represented for every sample. The expression patterns in colon and in brain tissues were similar. There was only very low to no measurable *Gsdme* expression in *Gsdme* KO mice, in contrast to WT mice where *Gsdme* expression was higher and more variable.

To confirm the *Gsdme* KO status of the generated mice, we performed *Gsdme* mRNA and protein expression analyses. *Gsdme* mRNA expression analyses on *Gsdme* KO (*n* = 7) and WT (*n* = 9) mice were performed, both on brain (*n* = 15) and colorectal (*n* = 16) tissues. For normalization, the most stable housekeeping genes were selected using geNorm (Table S1). *Gsdme* mRNA expression was statistically significantly lower in *Gsdme* KO mice compared to WT mice, both in brain (*p*-value = 0.00031) and in colorectal tissues (*p*-value = 0.00017) (Figure 1).

**Figure 2:**
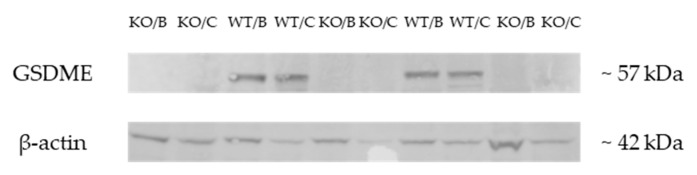
Representative example of a Western Blot on samples of *Gsdme* KO and WT mice. Western Blotting was performed with a primary rabbit anti-DFNA5/GSDME-N-Terminal antibody (ab215191) and a secondary Goat Anti-Rabbit IgG (H+L)-HRP Conjugate (#1706515). Clear GSDME protein bands (~57 kDa) were seen, both in the brain and colorectal tissue of WT mice. GSDME could not be detected in *Gsdme* KO brain or colorectal tissues. In all samples, clear bands for β-actin could be seen (~42 kDa). WT = wild type mice; KO = *Gsdme* knockout mice; B = brain tissue; C = colorectal tissue.

Besides mRNA expression analyses, Western Blotting was also performed. GSDME protein expression was compared between *Gsdme* KO (*n* = 7) and WT (*n* = 9) mice, both on brain (*n* = 16) and colorectal tissues (*n* = 16) to confirm the GSDME KO status of the mice generated. As shown in Figure 2, GSDME protein expression was completely absent in *Gsdme* KO brain and colorectal tissues. These results confirm that the mice used for this experiment were *Gsdme* KO mice.

### 2.2. Chemically Induced Intestinal Cancer Mouse Model

**Figure 3:**
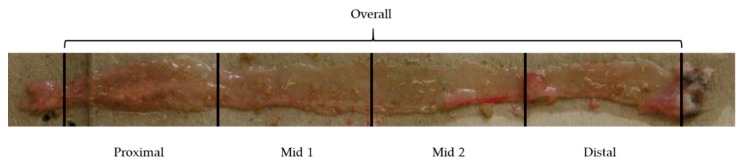
Large intestine of a representative mouse. The large intestine was opened longitudinally and divided into four parts: proximal, mid 1, mid 2, and distal.

We hypothesized that *Gsdme* KO mice are more prone to the development of proliferative lesions compared to WT mice. To investigate this hypothesis we induced colorectal neoplasia by injections of AOM, both in *Gsdme* KO and WT mice. Three parallel experiments were performed (Table 2). After dissection, all mice were microscopically analyzed, paying special attention to the large intestine for the identification of proliferative lesions, and to the liver to evaluate possible metastases (Figure 3, Table 1 and Table S2).

#### 2.2.1. Intestinal Pathology in the Chemically Induced Model

A minor degree (minimal to slight) of mucosal inflammation by mononuclear cell infiltration was noted in most of the animals. In several mice, in some histology slides, the inflammation was more pronounced (moderate (*n* = 39/100) to marked (*n* = 5/100); Table 1). There was no statistically significant difference between *Gsdme* KO and WT mice for moderate mucosal inflammation (Table 1). Marked mucosal inflammation was significantly more present in *Gsdme* KO (*n* = 4/46) compared to WT (*n* = 1/54) mice (overall: *p*-value = 0.014; mid 2: *p*-value = 0.019) (Table 1). However, the number of mice with marked mucosal inflammation in the colon was rather low (*n* = 5). For moderate mucosal inflammation, a significant interaction between genotype and timepoint (age) was found (overall: *p*-value = 0.0091; mid 1: *p*-value = 0.0086; mid 2: *p*-value = 0.0052; distal: *p*-value = 0.025). This indicates that the effect of genotype on the presence of moderate mucosal inflammation was not the same across the different timepoints. Compared to *Gsdme* KO (*n* = 1/13), more WT (*n* = 10/24) mice had moderate mucosal inflammation in the large intestine at 20 weeks. This pattern switched at 22 weeks, where more *Gsdme* KO (*n* = 12/24) compared to WT (*n* = 3/13) mice showed moderate mucosal inflammation. This was also true for 24 weeks (*Gsdme* KO: *n* = 6/9; WT: *n* = 7/17; Table S2).

Mucosal edema of minor degree (minimal to slight) was observed in some mice, especially in the distal colon. Cysts of the glandular mucosa without cellular atypia were often seen, both in *Gsdme* KO and WT mice (Table 1). Those glandular cysts in the first half of the large intestine (proximal and mid 1), were statistically significantly more present in WT compared to *Gsdme* KO mice (proximal: *p*-value = 0.043; mid 1: *p*-value = 0.00038; Table 1 and Table S2).

##### Proliferative Lesions in the Chemically Induced Model

**Figure 4:**
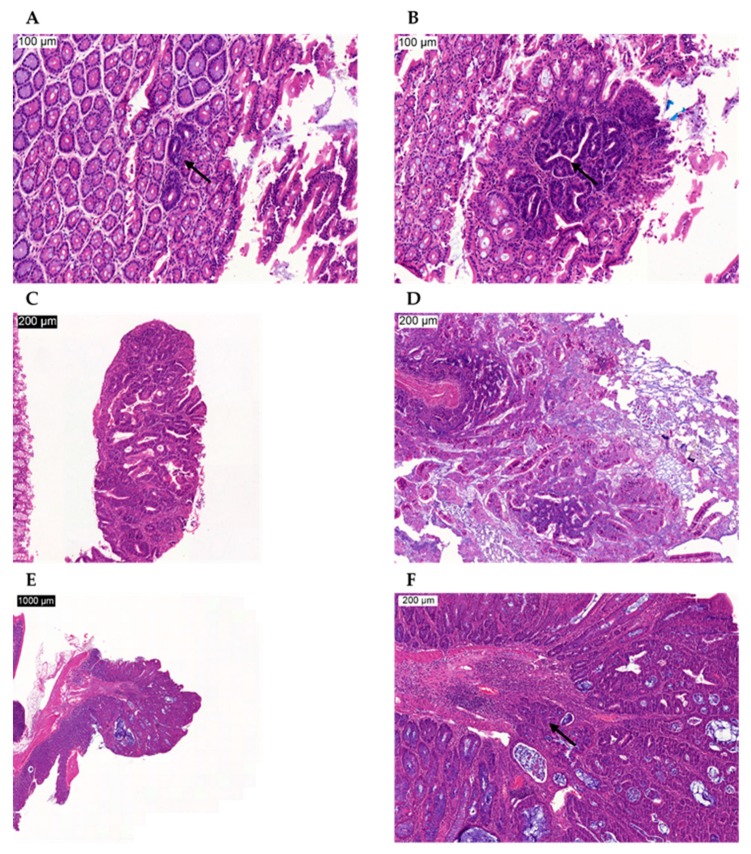
Microscopic images of proliferative lesions in AOM-treated mice. (**A**) Typical hyperplasia, indicated by the arrow, in the large intestine. (**B**) Atypical hyperplasia, indicated by the arrow, in the large intestine. (**C**) Adenoma in the large intestine. (**D**) Adenoma in the small intestine. (**E**) Adenocarcinoma in the large intestine. (**F**) Magnification of the adenocarcinoma in the large intestine (E). The arrow indicates infiltration of the adenocarcinoma in the submucosa. Scale bars are indicated on the images.

Because we wanted to determine the tumor-suppressive effects of GSDME, we were mainly interested in the proliferative lesions occurring in the large intestine [32]. The following proliferative lesions were found: typical hyperplasia, atypical hyperplasia, adenoma, and adenocarcinoma (Figure 4). Logistic regression analyses on the presence or absence of each of those lesions based on genotype, and accounting for sex and age, for the overall large intestine and for each of the four locations (proximal, mid 1, mid 2, and distal) were performed. Remarkably, no proliferative lesions were found in the most proximal part of the large intestine, neither in WT nor in *Gsdme* KO mice (Table 1). Moreover, the number of mice with adenoma was very limited (*n* = 4/100; Table 1; Figure 4C). In general, there were no statistically significant differences between *Gsdme* KO and WT mice for the presence of at least one lesion for each of those proliferative lesions (Table 1), except for atypical hyperplasia at location mid 1 (*p*-value = 0.023). There were only five animals with atypical hyperplasia at mid 1 and all of those mice were *Gsdme* KO (Table 1). For the lesions documented in Supplementary Table S3 (typical hyperplasia, adenocarcinoma, and proliferative lesion), a significant association with the sex of the mice was found. Remarkably, those lesions were significantly more present in female compared to male mice. Interaction terms of genotype × sex and genotype × timepoint (age) were never significant, meaning that the effect of genotype on the number of affected mice was not dependent on sex and that the rate at which *Gsdme* KO and WT mice developed proliferative lesions was not significantly different. We can conclude that there were no large differences in the number of mice with one or more proliferative lesions between *Gsdme* KO and WT mice.

Subsequently we analyzed the differences in the absolute number of lesions per mouse between *Gsdme* KO and WT mice by quasi-poison analyses, accounting for sex and age. No statistically significant differences between *Gsdme* KO and WT mice were found, except for the number of atypical hyperplasic lesions at mid 1, where there was a statistically significant effect for genotype (*p*-value = 7.36 × 10^−7^). The quasi-poison analysis thus reached the same conclusions as the logistic regression analysis.

(1) Tumor Characteristics in the Chemically Induced Model

**Figure 5:**
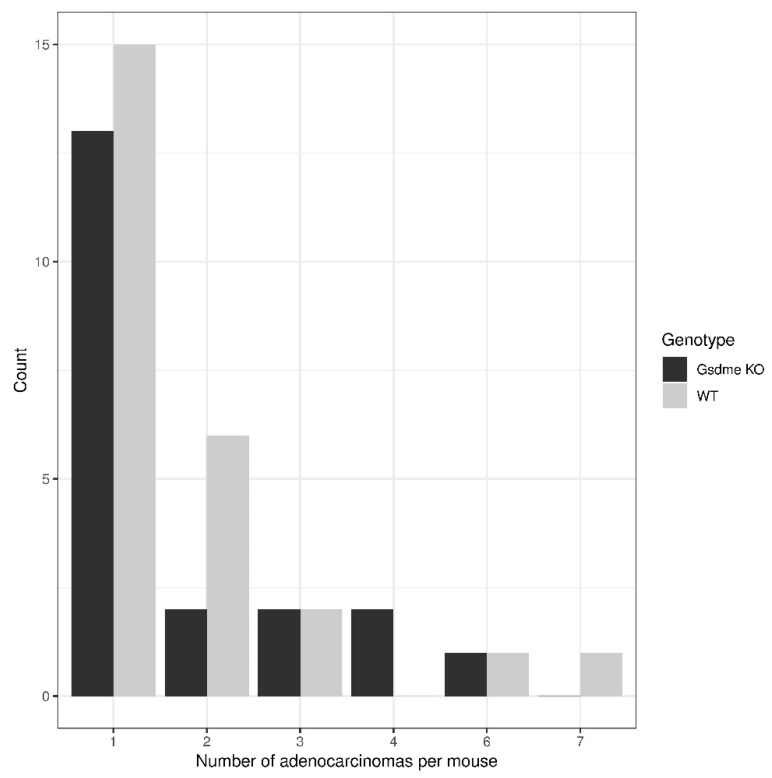
Distribution of the number of adenocarcinomas per mouse in *Gsdme* KO and WT mice. There were 20 out of 46 (43.5%) *Gsdme* KO and 25 out of 54 (46.3%) WT mice with one or more colon adenocarcinomas, with a median of one adenocarcinoma/mouse (range: 1–6) for the *Gsdme* KO mice and a median of one adenocarcinoma/mouse (range: 1–7) for the WT mice.

**Figure 6:**
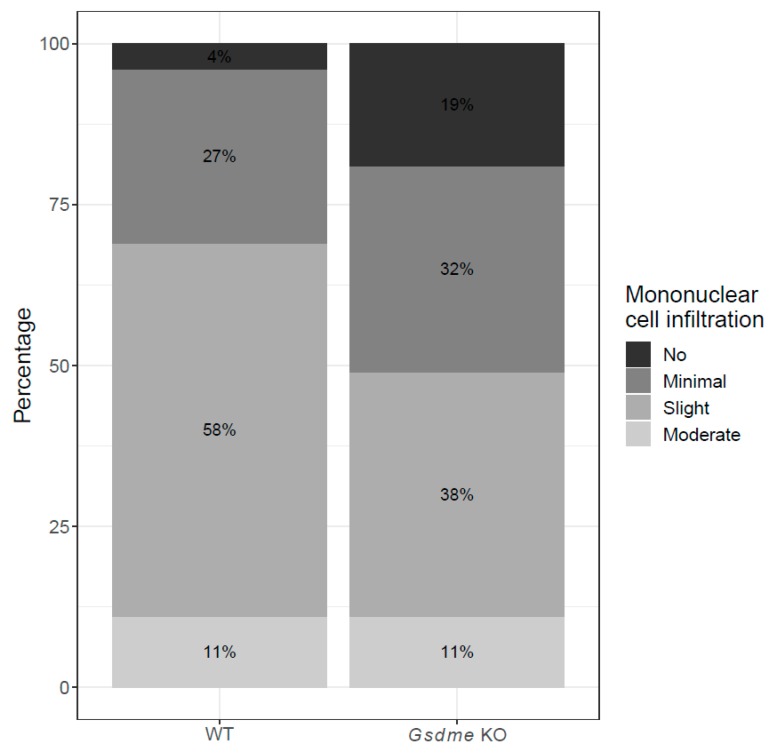
Percentage of adenocarcinomas with different grades of mononuclear cell infiltration in WT and *Gsdme* KO mice. Slight inflammation was more often associated with adenocarcinomas in WT mice compared to *Gsdme* KO mice, while in the latter group more often no inflammation was present.

There were 20 out of 46 (43.5%) *Gsdme* KO and 25 out of 54 (46.3%) WT mice with one or more colon adenocarcinomas (Figure 4E,F), with a median of one adenocarcinoma/mouse (range: 1–6) for the *Gsdme* KO mice and a median of one adenocarcinoma/mouse (range: 1–7) for the WT mice (Figure 5 and Table S4). For each of these adenocarcinomas, the following characteristics were scored: location, morphology, differentiation grade, grade of mononuclear cell infiltration, presence of fibrosis, and the number of slides in which the adenocarcinoma was seen (Table S4). Most of the adenocarcinomas were located in the distal part of the colon and were well-differentiated. The adenocarcinomas were frequently associated with mostly minimal to slight inflammatory cell infiltration. Fibrosis was present only in a few mice, but was significantly more present in WT (*n* = 9/46) compared to *Gsdme* KO (*n* = 2/37) adenocarcinomas (*p*-value = 0.048). Remarkably, there were more female mice (*n* = 30/54) with at least one adenocarcinoma throughout the large intestine, compared to male mice (*n* = 15/46; *p*-value = 0.024; Table S3). No additional statistically significant differences between WT and *Gsdme* KO mice for these tumor characteristics were found (Table S4). Nevertheless, a trend for the grade of mononuclear cell infiltration between WT and *Gsdme* KO mice was found (*p*-value = 0.056; Figure 6).

#### 2.2.2. Other Organs Analyzed in the Chemically Induced Model

Microscopic analyses of the liver of all 100 mice were performed. No metastases were found. Hepatic lesions that were found in several mice were characteristic of cirrhosis (Figure S1). The cirrhosis encountered here was probably related to the AOM treatment [33,34]. Other hepatic lesions found were most probably due to treatment with AOM or hemodynamic changes during sacrifice [33,34]. No statistically significant differences for any of these hepatic lesions were found between WT and *Gsdme* KO mice. For more detailed information on the hepatic lesions see Supplementary Material.

Lungs from 22 *Gsdme* KO and 16 WT mice were analyzed. No metastases were found in these mice. Pulmonary lesions that were present were most probably related to hemodynamic changes during sacrifice.

For the AOM-induced model, we were mainly interested in the proliferative lesions occurring in the large intestine. However, the small intestines of three WT and eight *Gsdme* KO mice were investigated. In one *Gsdme* KO mouse a slight to moderate focus of atypical hyperplasia was noted in the proximal part of the small intestine. In another *Gsdme* KO mouse, three proliferative lesions were found in the proximal part of the small intestine. Two focal, slight, and well-differentiated adenocarcinomas were found. In addition, one lesion of moderate, atypical hyperplasia was seen. Finally, one *Gsdme* KO mouse had an adenoma in the proximal part of the small intestine. There was one WT mouse with an adenocarcinoma in the proximal part of the small intestine.

### 2.3. Genetically Modified Intestinal Cancer Mouse Model

We hypothesized that *Gsdme* KO mice are more prone to the development of proliferative lesions compared to WT mice. To investigate this hypothesis we induced gastrointestinal neoplasia by crossing *Gsdme* KO mice with *Apc^1638N/+^* mice, resulting in *Apc^1638N/+^* *Gsdme* KO and *Apc^1638N/+^* *Gsdme* WT mice (Table 3). After dissection, all mice were microscopically analyzed, paying special attention to the small intestine, liver and lungs. In contrast to the AOM model, *Apc^1638N/+^* mice developed most of the adenocarcinomas in the small intestine (Table 1). Liver and lungs were analyzed to identify possible metastases.

#### 2.3.1. Intestinal Pathology in the Genetically Modified Model

Some minor degrees (minimal to slight) of mucosal inflammation by mononuclear cell infiltration were noted in most of the animals. In a few mice, in some histology slides, the inflammation was more pronounced (moderate) (Table 1). There were significantly more *Apc^1638N/+^ Gsdme* WT mice (*n* = 16/24), compared to *Apc^1638N/+^ Gsdme* KO mice (*n* = 3/13) with moderate mucosal inflammation throughout the small intestine (*p*-value = 0.010). Especially for moderate mucosal inflammation at mid 2, there was a statistically significant difference (*p*-value = 0.0088). There were seven animals with moderate mucosal inflammation and all of them were *Gsdme* WT mice (Table 1). Marked mucosal inflammation was never found (Table 1).

##### Proliferative Lesions in the Genetically Modified Model

As we wanted to investigate the tumor-suppressive effects of GSDME, we were mainly interested in the proliferative lesions occurring in the small intestine. The following proliferative lesions were found in the small intestine: typical hyperplasia, atypical hyperplasia, and adenocarcinoma (Table 1; Figure 4D). Logistic regression analyses on the presence or absence of each of these lesions based on genotype, and accounting for sex, for the overall small intestine and for each of the four locations (proximal, mid 1, mid 2, and distal) were performed. In general, most of the proliferative lesions were located in the proximal part of the small intestine (Table 1). Typical hyperplasia throughout the small intestine was significantly more present in *Apc^1638N/+^ Gsdme* WT (*n* = 9/24) compared to *Apc^1638N/+^ Gsdme* KO (*n* = 1/13) mice (*p*-value = 0.034; Table 1). This was especially true for the most proximal part of the small intestine (*p*-value = 0.0077; *n* = 7/24 WT; *n* = 0/13 KO). At mid 2 of the small intestine there were four mice with one or more adenocarcinomas, and all of these mice had an *Apc^1638N/+^ Gsdme* WT genotype (*p*-value = 0.049; Table 1). Remarkably, no adenomas were found, neither in the *Apc^1638N/+^ Gsdme* WT nor in the *Apc^1638N/+^ Gsdme* KO mice (Table 1). For adenocarcinomas and proliferative lesions in total, located in the proximal part of the small intestine, significant associations with the sex of the mice were found (*p*-value = 0.0092 and *p*-value = 0.023, respectively). Remarkably, these lesions were significantly more present in male compared to female mice, in contrast to what was found in the chemically induced model.

Subsequently, we analyzed the differences in the absolute number of lesions per mouse between *Apc^1638N/+^ Gsdme* KO and *Apc^1638N/+^ Gsdme* WT mice by quasi-poison analyses, accounting for sex. The same significant associations as with logistic regression analysis were found (number of typical hyperplastic lesions overall (*p*-value = 0.029), and at the proximal part of the small intestine (*p*-value = 0.00045); number of adenocarcinomas at mid 2 (*p*-value = 0.012)). The same conclusions as with logistic regression analysis can be made.

(1) Tumor characteristics in the genetically modified model

**Figure 7:**
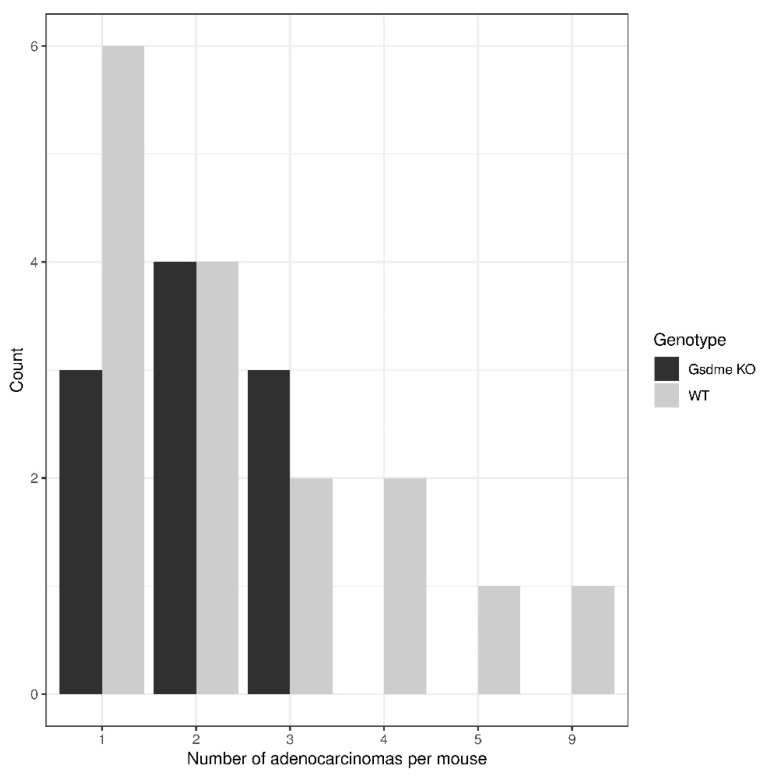
Distribution of the number of adenocarcinomas per mouse in *Apc^1638N/+^ Gsdme* KO and *Apc^1638N/+^ Gsdme* WT mice. There were 10 out of 13 (76.9%) *Apc^1638N/+^ Gsdme* KO and 16 out of 24 (66.7%) *Apc^1638N/+^ Gsdme* WT mice with one or more adenocarcinomas in the small intestine, with a median of two adenocarcinomas/mouse (range: 1–3) for the *Gsdme* KO mice, and a median of two adenocarcinomas/mouse (range: 1–9) for the WT mice.

There were 10 out of 13 (76.9%) *Apc^1638N/+^ Gsdme* KO and 16 out of 24 (66.7%) *Apc^1638N/+^ Gsdme* WT mice with one or more adenocarcinomas in the small intestine, with a median of two adenocarcinomas/mouse (range: 1–3) for the *Gsdme* KO mice and a median of two adenocarcinomas/mouse (range: 1–9) for the WT mice (Figure 7 and Table S5). For each of those adenocarcinomas, the following characteristics were scored: location, morphology, grade of mononuclear cell infiltration and presence of fibrosis (Table S5). Most of the adenocarcinomas were located in the proximal part of the small intestine and all were well-differentiated. The adenocarcinomas were frequently associated with mostly minimal to slight inflammatory cell infiltration. Fibrosis was present only in a few mice. There were no statistically significant differences in tumor characteristics between *Apc^1638N/+^ Gsdme* KO and *Apc^1638N/+^ Gsdme* WT adenocarcinomas (Table S5).

#### 2.3.2. Other Organs Analyzed in the Genetically Modified Model

Microscopic analyses of the liver and lungs of all 37 mice were performed. No metastases were found. The identified hepatic and pulmonary lesions were most probably related to circulation disorders during sacrifice. In this model no cirrhosis was seen, which supports the finding that the cirrhosis observed in AOM-treated mice was probably related to the AOM treatment. None of these lesions were significantly different between *Apc^1638N/+^ Gsdme* WT and *Apc^1638N/+^ Gsdme* KO mice. For more detailed information on the hepatic and pulmonary lesions, see Supplementary Material.

## 3. Discussion

In this study, we evaluated the tumor-suppressive effects of GSDME in two intestinal cancer mouse models. Spontaneous intestinal tumors in laboratory mice are rare [35]. Therefore we opted to promote tumor initiation in *Gsdme* KO and WT mice either by i.p. injections with AOM or by crossing them with *Apc^1638N/+^* mice. The incidence of colonic adenocarcinomas after injection of AOM is highly dependent on the mouse strain used [18,36]. The *Gsdme* KO mice have a C57BL/6N background, which has an incidence of colon adenocarcinomas of ~50%, with 1.0 ± 1.2 lesions per mouse after treatment with AOM and dextran sodium sulfate (DSS), by 23 weeks of age [36]. Hence, it was possible to analyze the effects of GSDME on the incidence and multiplicity of colon adenocarcinomas. In our study, the overall incidence and the median multiplicity of colon adenocarcinomas in WT mice, treated with AOM, closely resembles previous studies in C57BL/6N mice [36]. Nevertheless, we did not detect any significant differences in incidence and median multiplicity with *Gsdme* KO mice. In contrast to what is generally reported in the literature, significantly more female, compared to male mice, had at least one adenocarcinoma throughout the large intestine in our study [37,38]. In general, AOM mouse models lack mucosal invasiveness and metastasis is rare [18,19,39,40]. In our study, metastases to the liver were not seen. Furthermore, we did not detect any significant difference for the overall incidence of proliferative lesions. The only proliferative lesion that showed a statistically significant difference between *Gsdme* KO and WT mice was atypical hyperplasia. If all timepoints were considered together, only five mice had atypical hyperplasia at mid 1 and they all had a *Gsdme* KO genotype. If we analyze the data per timepoint, a significant difference in the incidence of atypical hyperplasia could be found at 22 weeks. There were only nine mice that had atypical hyperplasia in the colon at this timepoint and all these mice had a *Gsdme* KO genotype. We also analyzed the multiplicity of the proliferative lesions in the colon. Besides the difference in atypical hyperplasia mentioned above, we did not find any additional significant differences in the multiplicity of proliferative lesions between *Gsdme* KO and WT mice. AOM is known to induce tumors in the distal part of the colon, which is also the predominant localization of spontaneous colorectal cancer in humans [18,19,22]. In our study, most of the proliferative lesions were also located in the distal part of the colon. In the most proximal part, no proliferative lesions were detected. Remarkably, a colon adenoma was only detected in 4% of the mice. This is in large contrast to the study by Suzuki et al., which described an incidence of adenomas of 70% in 23 week old C57BL/6N mice [36]. However, in the latter study mice were treated with DSS, in addition to AOM. In the study by Tanaka et al. [41], the incidence of adenomas varied between 0% and 38%, depending on the treatment regimen (AOM and/or DSS). In five, 25 week old, Crj: CD-1 male mice that received a single i.p. injection of AOM, no adenomas were present [41]. Finally, in our study, the rate at which *Gsdme* KO and WT mice developed proliferative lesions was not significantly different.

Next, we analyzed the role of GSDME in an *Apc^1638N/+^* mouse model; because we were mainly interested in proliferative lesions, we focused on the small intestine in this experiment. Similarly to the study by Fodde et al. [21], the proliferative lesions found in our study were also mainly located in the proximal part of the small intestine. Typical hyperplasia was significantly more present in *Apc^1638N/+^ Gsdme* WT compared to *Apc^1638N/+^ Gsdme* KO mice. The difference was most pronounced in the most proximal part of the small intestine, where seven mice showed typical hyperplasia and all of them were *Apc^1638N/+^ Gsdme* WT. Atypical hyperplasia was found in both *Apc^1638N/+^ Gsdme* WT and *Apc^1638N/+^ Gsdme* KO mice, but no difference in incidence was found. Remarkably, in our study, no adenomas were observed. This is in contrast to the original study by Fodde et al. [21], where eight *Apc^1638N/+^* mice were analyzed before 22 weeks of age. Three out of eight of these mice had at least one adenoma in the small intestine. We cannot see a reasonable explanation for this finding. At 22 weeks of age, 76.9% of *Gsdme* KO and 66.7% of WT mice had developed at least one adenocarcinoma in the small intestine. This is in line with the study by Fodde et al., where five out of eight (62.5%) mice had at least one adenocarcinoma, all located in the small intestine [21]. However, the incidence of adenocarcinomas in *Apc^1638N/+^ Gsdme* KO mice was not significantly higher compared to the incidence in *Apc^1638N/+^ Gsdme* WT mice. The only significant difference between both genotypes was at mid 2, where there were only four mice with an adenocarcinoma and all of them were *Apc^1638N/+^ Gsdme* WT. No metastases to the liver or lungs were observed. The study by Fodde et al. (1994) reported metastasis to the liver in one *Apc^1638N/+^* mouse. However, this mouse was already 12 months old [21]. The mean multiplicity of adenocarcinomas in the study by Fodde et al. [21] and in our study was two, both for *Apc^1638N/+^ Gsdme* WT and *Apc^1638N/+^ Gsdme* KO mice. When all types of proliferative lesions in the small intestine were considered together, 95.8% of *Apc^1638N/+^ Gsdme* WT and 84.6% of *Apc^1638N/+^ Gsdme* KO had one or more proliferative lesions. In the study by Fodde et al. this was 75.0%, but this was only on eight *Apc^1638N/+^* mice and they only considered the neoplastic lesions (adenomas and adenocarcinomas) [21]. From both the chemical and genetic experiments, we can conclude that there were no major differences between *Gsdme* KO and WT mice for the number of mice with at least one adenocarcinoma or in the number of proliferative lesions or adenocarcinomas per mouse.

There are several possible explanations why no major differences were observed in our current study. Firstly, the susceptibility to tumor development is highly dependent on the genetic background of the mouse strain used [18,36], it is possible that our two models are not suitable to detect these differences. Possibly, the effect of GSDME on tumorigenesis is masked by the effect of AOM or *Apc* in these models. Furthermore, mice were sacrificed at 20, 22, or 24 weeks of age, and it is possible that at a later tumor progression stage, differences between *Gsdme* KO and WT mice would become apparent. On the other hand, it is also possible that the role of GSDME in tumorigenesis is more subtle than anticipated. Support for this notion can be found in the recent developments on the functional role of GSDME. These new findings indicate that GSDME acts as an executioner of necrotic cell death instead of an inducer of programmed cell death, as previously thought.

Recent breakthroughs in unraveling the function of gasdermins showed that the GSDME protein is activated through cleavage by caspase-3 [42,43]. During this process, an active and toxic N-GSDME fragment is formed that targets the plasma membrane and permeabilizes it by pore formation, resulting in secondary necrosis or pyroptosis. Secondary necrosis is a lytic and inflammatory phase that occurs when apoptotic cells are not scavenged [42]. Pyroptosis, which is also a form of regulated necrosis, is a lytic type of cell death inherently associated with infection and inflammation [43]. Soon after these studies, several other studies pointed towards an important role for GSDME in secondary necrosis and its possible pathophysiological and therapeutic implications, especially in cancer [44,45,46,47,48,49,50]. These very recent findings changed the view on the expected role of GSDME in cancer. When we started the study reported in this paper, GSDME was thought to be an inducer of programmed cell death [2,3,4]. Therefore, we hypothesized that *GSDME* was a tumor suppressor gene and by knocking out this gene in mice we expected more affected *GSDME* KO mice and more adenocarcinomas in the *GSDME* KO compared to the WT mice. However, since the breakthrough on gasdermin function, we expect that the effects of GSDME in tumor biology will probably be more subtle. It is possible that the importance of GSDME may be in creating an inflammatory microenvironment around the tumor, by induction of necrosis or pyroptosis. This is in line with what we found in our study; moderate mucosal inflammation in the small intestine was more often present in *Apc^1638N/+^ Gsdme* WT compared to *Apc^1638N/+^ Gsdme* KO mice, especially at mid 2 in the small intestine, where only *Apc^1638N/+^ Gsdme* WT showed moderate mucosal inflammation. This is what we expected, given the currently known function of GSDME. When GSDME is present (WT), cells can go into secondary necrosis or pyroptosis. When GSDME is not present (KO), cells stay in the apoptotic phase, which does not induce an inflammatory reaction [51]. In the chemical experiment, no statistically significant difference between *Gsdme* KO and WT mice, with respect to moderate inflammation, was found if we consider all timepoints together. However, this observation seems time dependent, because at 20 weeks there was a strong trend towards more WT mice with moderate inflammation throughout the colon compared to *Gsdme* KO mice, while at timepoint 22 weeks and 24 weeks there was an inverse trend towards more *Gsdme* KO mice with moderate inflammation compared to WT mice. This contradiction makes it difficult to draw a clear conclusion for the chemical experiment. If we look specifically at inflammation associated with adenocarcinomas, we were able to find a clear trend showing a more severe grade of mononuclear cell infiltration in the *Gsdme* WT adenocarcinomas when compared to the *Gsdme* KO adenocarcinomas in the chemical experiment. In the *Gsdme* KO adenocarcinomas more often no inflammation was noted, which is in line with our proposed hypothesis.

In our study there was no statistically significant difference in the number of mice with one or more adenocarcinomas, or in the number of mice with one or more proliferative lesions between *Gsdme* KO and WT mice. Moreover, there was no statistically significant difference in the number of lesions per mouse between *Gsdme* KO and WT mice. Due to the recent progress in unravelling the function of gasdermins, we speculate that GSDME may have a more subtle effect in tumor biology by creating a more inflammatory microenvironment. However, more research is needed to further unravel the exact function of GSDME in cancer.

## 4. Materials and Methods

All mice were maintained at the Animal Facility of the University of Antwerp according to the institutional animal care guidelines, and were maintained under controlled conditions of humidity (45–65%), light (12 h light/12 h dark cycle), and temperature (20–24 °C). The mice were offered tap water and ssniff^®^ R/M-H pelleted diet (ssniff-Spezialdiäten GmbH, Soest, Germany) ad libitum.

This study was approved by the Ethical Committee for Animal Testing (ECD) of the University of Antwerp (permission number: 2014-40).

### 4.1. Gsdme Knockout Mouse

A male chimeric *Gsdme* KO mouse, *Gsdme^tm1a(KOMP)Wtsi^*, was designed in 2011 by KOMP (Knockout Mouse Project; University of California, Davis, CA 95616, USA). This mouse was crossed with a female WT C57BL/6N mouse (Charles River Laboratories [LA2230391], Wilmington, MA, USA) to obtain a male heterozygous *Gsdme* KO mouse. This mouse was crossed with female WT C57BL/6N mice to obtain more heterozygous *Gsdme* KO mice. In turn, these heterozygous *Gsdme* KO mice were crossed to obtain *Gsdme* KO and WT mice.

When mice were four weeks old, they were weaned and ear punched. DNA was extracted from the ear punches (MyTaq™ Extract-PCR Kit, Bioline, Memphis, TN, USA) and mice were genotyped by PCR. The genotyping protocol for both *Gsdme* and *Apc^1638N^* can be found in the Supplementary Material (Tables S6–S11).

Mice were screened twice a week for abnormal behavior, weight loss, diarrhea, rectal bleeding and prolapse. If mice had lost ≥ 20% of their body weight, they were sacrificed.

#### 4.1.1. Chemically Induced Intestinal Cancer Mouse Model

Colorectal neoplasia were induced by injections of AOM, both in *Gsdme* KO and WT mice. Three parallel experiments were performed (Table 2). At the age of six weeks, baseline weights were recorded and all mice, both *Gsdme* KO and WT, received a first i.p. injection of AOM (10 mg/kg body weight; 13.4M, Sigma, Saint Louis, MO, USA), which was repeated weekly during four weeks. Mice were sacrificed using CO_2_, at different ages (20 weeks (*n* = 37), 22 weeks (*n* = 37), or 24 weeks (*n* = 26); Table 2).

#### 4.1.2. Genetically Modified Intestinal Cancer Mouse Model

We first bred male *Apc^1638N/+^* mice (kindly provided by Dr. Riccardo Fodde, Laboratory for Stem Cell and Cancer Research, Erasmus MC, Rotterdam, The Netherlands) with female WT C57BL/6J mice and subsequently outcrossed *Apc^1638N/+^* mice with *Gsdme* KO mice, leading to *Apc^1638N/+^ Gsdme^+/−^* mice. In turn, these heterozygous mice were intercrossed to obtain *Apc^1638N/+^ Gsdme* KO (*n* = 13) and *Apc^1638N/+^ Gsdme* WT (*n* = 24 mice), which were used for the experiment (Table 3). All mice were sacrificed using CO_2_ at 22 weeks of age.

### 4.2. Dissection and Histology

At the end of the study (20, 22, or 24 weeks of age), mice were sacrificed and examined macroscopically, paying special attention to the gastrointestinal tract (stomach, small intestine, and large intestine), liver, and lungs. The large intestines were rinsed with saline and opened longitudinally (Figure 3). Tissues were fixed in 10% buffered formalin for at least 24 h, and processed into paraffin blocks using routine procedures (stomach (1 FFPE block), small intestine (4 FFPE blocks—swiss rolls: proximal, mid 1, mid 2 and distal), large intestine (4 FFPE blocks—proximal, mid 1, mid 2, and distal), liver (1 or 2 FFPE blocks depending on the size), lungs (1 FFPE block), and eventually a macroscopic abnormality). From each FFPE block, five sections of 5µm were made every 100 µm (every 50 µm for the large intestine). The most distal part of the large intestine was fully cut. All sections were hematoxylin and eosin (HE) stained for microscopical examination by a veterinary pathologist (Dr. I. Debyser). Mouse genotypes were unknown to the investigating pathologist.

For the chemically induced experiment, the sections of the large intestine and liver were examined for all animals. For the genetic experiment the sections of the small intestine, liver and lungs were examined for all animals. For histological analyses, the microscopic findings were either graded (minimal, slight, moderate, or marked) or indicated as present or absent without grade. Intestinal tumors were scored according to the WHO nomenclature for histological assessment of intestinal tumors for mice and rodents [32,51].

### 4.3. qRT-PCR

The brain and large intestine were isolated from 22 week old WT (*n* = 8 [brain], *n* = 9 [large intestine]) and *Gsdme* KO (*n* = 7 (brain); *n* = 7 (large intestine)) C57BL/6N mice and mechanically homogenized using gentle MACS M Tubes (Miltenyi Biotec, Bergisch Gladbach, Germany) on a Dispomix^®^ (Wilten Instrumenten, Etten-Leur, Nederland). After homogenization, total RNA was extracted using the RNeasy kit (Qiagen, Hilden, Germany) according to the manufacturer’s instructions. Real-time PCR was performed using the qPCR MasterMix Plus for SYBR^®^ Green I No ROX (Eurogentec, Luik, Belgium) according to the manufacturer’s instructions, with the following primers: 5′AGCTCTTTGCAACAGCCTACTTCC3′ (forward; exon 8) and 5′TGTGGCATTATCAGGCATTTCTGC3′ (reverse; exons 8–9) on a Lightcycler 480 instrument (Roche, Basel, Switzerland). For the reference genes used for normalization, see Supplementary Table S1. The resulting gene expression data were analyzed using Qbase plus (Biogazelle, Gent, Belgium) software.

### 4.4. Western Blot

The brain and large intestine were isolated from 22 week old WT (*n* = 9 (brain), *n* = 9 (large intestine)) and *Gsdme* KO (*n* = 7 (brain), *n* = 7 (large intestine)) C57BL/6N mice and mechanically homogenized using gentleMACS M Tubes (Miltenyi Biotec) on a Dispomix^®^ (Wilten Instrumenten). Total protein was extracted from the homogenate using ice cold RIPA lysis and extraction buffer (Thermo Fisher Scientific, Waltham, MA, USA) and cOmplete™, Mini, EDTA-free Protease Inhibitor Cocktail (Roche, Basel, Switzerland). The protein lysate was diluted in NuPAGE™ LDS Sample Buffer and NuPAGE™ Sample Reducing Agent was added. The lysate was heated for 10 min at 70 °C, loaded on a NuPAGE™ 4–12% Bis-Tris Gel (Invitrogen, Carlsbad, CA, USA) and electrophoretically separated. Next, the membrane was incubated with a primary rabbit anti-DFNA5/GSDME-N-Terminal antibody (ab215191, 1/5000, Abcam, Cambridge, UK) and a secondary Goat Anti-Rabbit IgG (H + L)-HRP Conjugate (#1706515, 1/10,000, Biorad, Hercules, CA, USA). Subsequently, the antibodies were detected using ECL™ Prime Western Blotting Detection Reagent (GE Healthcare, Chicago, IL, USA) and visualized using the ImageQuant LAS 4000 mini (GE Healthcare). For β-actin detection, monoclonal mouse anti-β-actin antibody was used (A5316; Sigma, Saint Louis, MO, USA).

### 4.5. Statistical Analyses

The proportion of mice bearing microscopic proliferative lesions and the number and type of lesions per mouse were compared between *Gsdme* KO and WT mice, using respectively logistic and quasi-poison regression models. All analyses were performed per location in the intestine (proximal, mid 1, mid 2, and distal) and across the whole intestine (overall). Because males and females were not equally present in the WT and KO groups, we accounted for sex in the regression analyses. Moreover, timepoint (age at sacrifice) was also included as a covariate to evaluate the effect of age at sacrifice on the presence of lesions. Furthermore, the histopathological features of the adenocarcinomas were collected and analyzed by either chi-square or Mann–Whitney *U* tests. *p*-values for the association between the grade of mononuclear cell infiltration and genotype were obtained via Armitage’s trend test with Monte Carlo simulation. To test differences in *Gsdme* mRNA expression between WT and KO mice, a Wilcoxon signed-rank test was used. All statistical analyses were carried out using the statistical package R, version 3.5.1 [52] or SPSS software (version 25; SPSS Inc., Chicago, IL, USA). All *p*-values are two-sided and *p*-values ≤ 0.05 were considered statistically significant.

## 5. Conclusions

When we initiated this study, GSDME was thought to be an inducer of programmed cell death. Moreover, *GSDME* was frequently epigenetically silenced in different cancer types. Therefore, we aimed to evaluate the tumor-suppressive effects of GSDME in two intestinal cancer mouse models. To mimic the silencing of *GSDME* by methylation, observed in human cancers, a *Gsdme* KO mouse was developed. The effects of GSDME on tumorigenesis were studied both in a chemically induced and in a genetic intestinal cancer mouse model. We expected more affected *Gsdme* KO mice and more adenocarcinomas or proliferative lesions in the *Gsdme* KO compared to the WT mice. However, we did not find any major differences between *Gsdme* KO and WT mice, neither for the number of affected mice nor for the multiplicity of proliferative lesions per mouse. Nevertheless, an important finding in line with the current literature on the function of gadermins, was a trend towards more severe inflammation in the *Gsdme* WT compared to *Gsdme* KO mice. We hypothesize that GSDME plays a role in creating a more inflammatory microenvironment around the tumor, by induction of necrosis or pyroptosis. We conclude that the effects of GSDME in tumor biology are probably more subtle than previously thought.

## Figures and Tables

**Table 1:** Overview of all lesions scored in the large intestine of AOM-treated mice (all timepoints combined) and in the small intestine of *Apc^1638N/+^* mice.

Lesion	Chemical Experiment—Large Intestine				Genetic Experiment—Small Intestine			
	*Gsdme* KO (*n* = 46)	WT (*n* = 54)	Δ (%)	*p*-Value	*Apc^1638N/+^ Gsdme* KO (*n* = 13)	*Apc^1638N/+^ Gsdme* WT (*n* = 24)	Δ (%)	*p*-Value
OVERALL								
moderate mucosal inflammation	19	20	4.3	0.73	3	16	−43.6	**0.010**
marked mucosal inflammation	4	1	6.8	**0.014**	0	0	0.0	-
glandular cyst	46	54	0.0	-	0	0	0.0	-
typical hyperplasia	21	26	−2.4	0.70	1	9	−29.8	**0.034**
atypical hyperplasia	18	13	15.0	0.081	5	9	1.0	0.90
adenoma	3	1	4.6	0.57	0	0	0.0	-
adenocarcinoma	20	25	−2.8	0.67	10	16	10.2	0.55
proliferative change	34	36	7.2	0.22	11	23	−11.2	0.21
PROXIMAL								
moderate mucosal inflammation	6	4	5.6	0.31	2	9	−22.1	0.13
marked mucosal inflammation	0	0	0.0	-	0	0	0.0	-
glandular cyst	25	43	−25.3	**0.043**	0	0	0.0	-
typical hyperplasia	0	0	0.0	-	0	7	−29.2	**0.0077**
atypical hyperplasia	0	0	0.0	-	3	6	−1.9	0.97
adenoma	0	0	0.0	-	0	0	0.00	-
adenocarcinoma	0	0	0.0	-	8	13	7.3	0.74
proliferative change	0	0	0.0	-	10	22	−14.8	0.17
MID 1								
moderate mucosal inflammation	9	7	6.6	0.37	1	5	−13.1	0.30
marked mucosal inflammation	1	0	2.2	0.053	0	0	0.0	-
glandular cyst	21	47	−41.3	**0.00038**	0	0	0.0	-
typical hyperplasia	2	4	−3.1	0.64	0	2	−8.3	0.19
atypical hyperplasia	5	0	10.9	**0.023**	1	5	−13.1	0.29
adenoma	0	0	0.0	-	0	0	0.0	-
adenocarcinoma	1	2	−1.5	0.69	5	8	5.2	0.77
proliferative change	8	6	6.3	0.53	6	12	−3.8	0.83
MID 2								
moderate mucosal inflammation	12	12	3.9	0.57	0	7	−29.2	**0.0088**
marked mucosal inflammation	3	0	6.5	**0.019**	0	0	0.0	-
glandular cyst	35	44	−5.4	0.79	0	0	0.0	-
typical hyperplasia	8	12	−4.8	0.25	0	2	−8.3	0.19
atypical hyperplasia	9	6	8.5	0.27	2	1	11.2	0.24
adenoma	1	0	2.2	0.50	0	0	0.0	-
adenocarcinoma	12	13	2.0	0.68	0	4	−16.7	**0.049**
proliferative change	24	21	13.3	0.20	2	6	−9.6	0.48
DISTAL								
moderate mucosal inflammation	7	11	−5.2	0.51	0	3	−12.5	0.098
marked mucosal inflammation	0	1	−1.9	0.49	0	0	0.0	-
glandular cyst	43	53	−4.7	0.57	0	0	0.0	-
typical hyperplasia	18	19	3.9	0.19	1	0	7.7	0.15
atypical hyperplasia	7	10	−3.3	0.95	0	0	0.0	-
adenoma	2	1	2.5	0.72	0	0	0.0	-
adenocarcinoma	13	18	−5.1	0.46	3	3	10.6	0.43
proliferative change	28	32	1.6	0.63	4	3	18.3	0.20

The number of mice with at least one specific lesion throughout the whole large intestine (chemical experiment) or small intestine (genetic experiment) (overall), or in one specific part of the large intestine/small intestine (proximal, mid 1, mid 2, and distal), in the *Gsdme* KO and WT group is indicated. The *p*-value indicates the significance for a difference between *Gsdme* KO and WT mice, accounting for sex and age. Proliferative change includes typical hyperplasia, atypical hyperplasia, adenoma, and/or adenocarcinoma.

**Table 2:** The number of mice per timepoint, with genotype and sex, used in the AOM-induced cancer mouse model experiment.

**Overall: 100 Mice**			
**46 *Gsdme* KO**		**54 WT**	
31 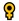	15 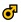	23 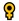	31 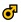
**20 Weeks: 37 Mice**			
**13 *Gsdme* KO**		**24 WT**	
9 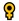	4 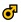	10 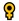	14 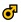
**22 Weeks: 37 Mice**			
**24 *Gsdme* KO**		**13 WT**	
16 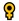	8 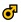	4 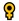	9 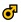
**24 Weeks: 26 Mice**			
**9 *Gsdme* KO**		**17 WT**	
7 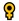	2 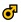	9 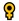	8 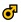

**Table 3:** The number of mice per genotype and sex, used in the *Apc^1638N/+^* cancer mouse model experiment.

Overall: 37 Mice			
13 *Apc^1638N/+^ Gsdme* KO		24 *Apc^1638N/+^ Gsdme* WT	
8 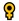	5 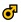	16 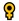	8 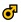

## Supplementary Materials

The following are available online at https://www.mdpi.com/2072-6694/11/8/1214/s1, Table S1: Reference genes used for normalization of *Gsdme* expression in brain and colon tissue, Table S2: Overview of all lesions scored in the large intestine of AOM-treated mice, sacrificed at respectively 20, 22 or 24 weeks of age, Table S3: Lesions that show a statistically significant association with the sex of the mice, Table S4: Overview of the adenocarcinoma characteristics in the AOM-treated mice, Table S5: Overview of the adenocarcinoma characteristics in the *Apc^1638N/+^ Gsdme* KO and *Apc^1638N/+^ Gsdme* WT mice, Table S6: Primers for *Gsdme* genotyping, Table S7: Mastermix for the *Gsdme* genotyping PCR, Table S8: Cycling parameters *Gsdme* genotyping PCR, Table S9: Primers for *Apc^1638N^* genotyping, Table S10: Mastermix for the *Apc^1638N^* genotyping PCR, Table S11: Cycling parameters *Apc^1638N^* genotyping PCR, Figure S1: Pathologies in the liver of AOM-treated mice.
