# Supplementary material for: Determination of the Potential Tumor-Suppressive Effects of Gsdme in a Chemically Induced and in a Genetically Modified Intestinal Cancer Mouse Model
Source: Cancers (Basel). 2019 Aug 20;11(8):1214. doi: 10.3390/cancers11081214 (PMC6721630; doi:10.3390/cancers11081214)
Supplement: Supplementary file 1 [file cancers-11-01214-s001.pdf]

Supplementary Material: Determination of the Potential Tumor-Suppressive Effects of Gsdme in a Chemically Induced, Genetically Modified Intestinal Cancer Mouse Model

Lieselot Croes, Erik Fransen, Marieke Hylebos, Kimberly Buys, Christophe Hermans, Glenn Broeckx, Marc Peeters, Patrick Pauwels, Ken Op de Beeck and Guy Van Camp

Table S1. Reference genes used for normalization of Gsdme expression in brain and colon tissue.

**Reference Genes - Brain**	**M**	**CV**
Actin, beta, cytoplasmic (Actb)	0.3099	0.1161
Glyceraldehyde-3-phosphate dehydrogenase (Gapdh)	0.3514	0.1498
HIV TAT specific factor 1 (Htatsf1)	0.3236	0.1274
Ribosomal protein L13a (Rpl13a)	0.4040	0.1863
Average	0.3472	0.1449
**Reference Genes - Colon**	M	CV
Adaptor-related protein complex 3, delta 1 subunit (Ap3d1)	0.3246	0.1398
Glyceraldehyde-3-phosphate dehydrogenase (Gapdh)	0.3480	0.1441
HIV TAT specific factor 1 (Htatsf1)	0.3104	0.1163
Average	0.3277	0.1334

The geNorm expression stability value of the reference gene (M) and the coefficient of variation of the normalized reference gene relative quantities (CV) are shown for every reference gene. Good reference genes have a M < 0.5 and a CV < 0.2. All primers, except the ones for *Rpl13a* (forward primer: 5′-CACTCTGGAGGAGAAACGGAAGG-3′ and reverse primer: 5′-GCAGGCATGAGGCAAACAGTC-3′), were primer mixes from the Mouse geNormPLUS kit (Primerdesign, Chandler's Ford, United Kingdom).

Table S2. Overview of all lesions scored in the large intestine of AOM-treated mice, sacrificed at respectively 20, 22 or 24 weeks of age.

	**20 Weeks**			**22 Weeks**			**24 Weeks**		
	***Gsdme* KO (N = 13)**	**WT (N = 24)**	**Δ (%)**	***Gsdme* KO (N = 24)**	**WT (N = 13)**	**Δ (%)**	***Gsdme* KO (N = 9)**	**WT (N = 17)**	**Δ (%)**
**COLON overall**									
moderate mucosal inflammation	1	10	−34.0%	12	3	26.9%	6	7	25.5%
marked mucosal inflammation	0	0	0.0%	1	0	4.2%	3	1	27.5%
glandular cyst	13	24	0.0%	24	13	0.0%	9	17	0.0%
typical hyperplasia	8	11	15.7%	7	3	6.1%	6	12	−3.9%
atypical hyperplasia	6	8	12.8%	9	0	37.5%	3	5	3.9%
adenoma	0	0	0.0%	3	0	12.5%	0	1	−5.9%
adenocarcinoma	5	11	−7.4%	9	4	6.7%	6	10	7.8%
proliferative change	9	15	6.7%	16	5	28.2%	9	16	5.9%
**PROXIMAL**									
moderate mucosal inflammation	1	3	−4.8%	3	1	4.8%	2	0	22.2%
marked mucosal inflammation	0	0	0.0%	0	0	0.0%	0	0	0.0%
glandular cyst	7	19	−25.3%	10	9	−27.6%	8	15	0.7%
typical hyperplasia	0	0	0.0%	0	0	0.0%	0	0	0.0%
atypical hyperplasia	0	0	0.0%	0	0	0.0%	0	0	0.0%
adenoma	0	0	0.0%	0	0	0.0%	0	0	0.0%
adenocarcinoma	0	0	0.0%	0	0	0.0%	0	0	0.0%
proliferative change	0	0	0.0%	0	0	0.0%	0	0	0.0%
**MID 1**									
moderate mucosal inflammation	0	5	−20.8%	6	1	17.3%	3	1	27.5%
marked mucosal inflammation	0	0	0.0%	0	0	0.0%	1	0	11.1%
glandular cyst	7	23	−42.0%	7	10	−47.8%	7	14	−4.6%
typical hyperplasia	0	0	0.0%	1	0	4.2%	1	4	−12.4%
atypical hyperplasia	0	0	0.0%	3	0	12.5%	2	0	22.2%
adenoma	0	0	0.0%	0	0	0.0%	0	0	0.0%
adenocarcinoma	1	1	3.5%	0	0	0.0%	0	1	−5.9%
proliferative change	1	1	3.5%	4	0	16.7%	3	5	3.9%
**MID 2**									
moderate mucosal inflammation	0	6	−25.0%	6	2	9.6%	6	4	43.1%
marked mucosal inflammation	0	0	0.0%	1	0	4.2%	2	0	22.2%

	**20 Weeks**			**22 Weeks**			**24 Weeks**		
	***Gsdme* KO (N = 13)**	**WT (N = 24)**	**Δ (%)**	***Gsdme* KO (N = 24)**	**WT (N = 13)**	**Δ (%)**	***Gsdme* KO (N = 9)**	**WT (N = 17)**	**Δ (%)**
glandular cyst	11	23	−11.2%	16	6	20.5%	8	15	0.7%
typical hyperplasia	1	3	−4.8%	4	2	1.3%	3	7	−7.8%
atypical hyperplasia	2	3	2.9%	5	0	20.8%	2	3	4.6%
adenoma	0	0	0.0%	1	0	4.2%	0	0	0.0%
adenocarcinoma	4	6	5.8%	4	1	9.0%	4	6	9.2%
proliferative change	7	10	12.2%	10	2	26.3%	7	9	24.8%
**DISTAL**									
moderate mucosal inflammation	0	5	−20.8%	6	1	17.3%	1	5	−18.3%
marked mucosal inflammation	0	0	0.0%	0	0	0.0%	0	1	−5.9%
glandular cyst	13	24	0.0%	22	12	−0.6%	8	17	−11.1%
typical hyperplasia	7	10	12.2%	6	1	17.3%	5	8	8.5%
atypical hyperplasia	4	7	1.6%	3	0	12.5%	0	3	−17.6%
adenoma	0	0	0.0%	2	0	8.3%	0	1	−5.9%
adenocarcinoma	2	8	−17.9%	6	4	−5.8%	5	6	20.3%
proliferative change	9	14	10.9%	13	5	15.7%	6	13	−9.8%

The number of mice with at least one specific lesion throughout the whole large intestine at respectively 20, 22 or 24 weeks of age, or in one specific part of the large intestine (proximal, mid 1, mid 2, distal), in the *Gsdme* KO and in the WT group is indicated. Proliferative change includes: typical hyperplasia, atypical hyperplasia, adenoma and/or adenocarcinoma.

Table S3. Lesions that show a statistically significant association with the sex of the mice.

**Lesion**	**Location**	**Number of Females (N = 54)**	**Number of Males (N = 46)**	***p* Value**
typical hyperplasia	mid 2	16	4	0.0061
adenocarcinoma	overall	30	15	0.024
	mid 1	3	0	0.041
proliferative lesion	mid 1	12	2	0.021
	mid 2	33	12	0.0016

Table S4. Overview of the adenocarcinoma characteristics in the AOM-treated mice.

Mice	Genotype	Sex	Age (Weeks)	Number of Adenocarcinoma	Location	Morphology	Differentiation	Number of Slides Seen	Relative Number of Slides Seen	Associated with Mononuclear Cell Infiltration	Associated with Fibrosis
1	WT	male	20	1	distal	tubular	well	3	3/10	slight	no
2	WT	female	20	1	distal	tubular	well	4	4/10	slight	no
3	WT	male	20	1	mid 2	tubular	well	3	3/5	slight	no
				2	distal	tubular	well	3	3/11	slight	no
4	WT	male	20	1	mid 2	tubular	well	4	4/5	slight	yes
				2	mid 2	tubular	well	4	4/5	slight	yes
5	WT	female	20	1	mid 2	tubular	well	2	2/5	slight	no
				2	mid 2	tubular	well	1	1/5	slight	no
				3	distal	tubular	well	2	2/11	slight	no
6	WT	male	20	1	distal	tubular	well	2	2/10	slight	no
7	WT	female	20	1	mid 2	tubular	well	2	2/5	slight	no
8	WT	male	20	1	distal	tubular	well	2	2/10	minimal	no
9	WT	female	20	1	mid 1	tubular-papillary	well	4	4/5	minimal	no
10	WT	female	20	1	mid 2	tubular	well	2	2/5	minimal	no
				2	distal	tubular	well	2	2/10	slight	no
11	WT	female	20	1	mid 2	tubular	well	5	5/5	no	no
				2	distal	tubular	well	1	1/10	minimal	no
12	*Gsdme* KO	female	20	1	mid 2	tubular	well	2	2/5	no	no
				2	mid 2	tubular	well	1	1/5	no	no
13	*Gsdme* KO	female	20	1	distal	/	well	4	4/7	minimal	no
14	*Gsdme* KO	female	20	1	mid 1	tubular	well	3	3/5	minimal	no
				2	mid 2	tubular	well	2	2/5	minimal	no
				3	mid 2	tubular	well	2	2/5	minimal	no
				4	mid 2	tubular	well	1	1/5	minimal	no
				5	distal	tubular	well	2	2/11	minimal	no
				6	distal	tubular	well	1	1/11	slight	no
15	*Gsdme* KO	female	20	1	mid 2	tubular	well	3	3/5	minimal	yes
16	*Gsdme* KO	male	20	1	mid 2	tubular	well	4	4/5	no	no
17	WT	male	22	1	distal	tubular	well	3	3/12	minimal	no
18	WT	female	22	1	distal	tubular	well	7	7/13	slight	no

**Mice**	**Genotype**	**Sex**	**Age (Weeks)**	**Number of Adenocarcinoma**	**Location**	**Morphology**	**Differentiation**	**Number of Slides Seen**	**Relative Number of Slides Seen**	**Associated with Mononuclear Cell Infiltration**	**Associated with Fibrosis**
19	WT	female	22	1	distal	tubular	well	7	7/10	slight	no
20	WT	male	22	1	mid 2	tubular	well	2	2/5	moderate	no
				2	distal	tubular	well	11	11/11	slight	no
				3	distal	tubular	well	9	9/11	slight	no
				4	distal	tubular	well	5	5/12	moderate	no
				5	distal	tubular	/	4	4/12	slight	no
				6	distal	tubular	/	4	4/12	slight	no
21	*Gsdme* KO	male	22	1	distal	tubular	well	10	10/10	slight	no
22	*Gsdme* KO	male	22	1	mid 2	tubular	moderate	3	3/5	moderate	no
				2	distal	tubular	moderate	3	3/9	moderate	no
				3	distal	tubular	well	2	2/9	no	no
23	*Gsdme* KO	female	22	1	distal	tubular-papillary	/	11	11/19	slight	no
24	*Gsdme* KO	female	22	1	mid 2	tubular	moderate	5	5/6	minimal	no
25	*Gsdme* KO	female	22	1	mid 2	tubular	well	5	5/5	slight	no
26	*Gsdme* KO	female	22	1	distal	tubular	well	3	3/11	slight	no
27	*Gsdme* KO	female	22	1	mid 2	tubular	well	3	3/5	moderate	no
28	*Gsdme* KO	female	22	1	distal	tubular	well	10	10/10	slight	no
29	*Gsdme* KO	female	22	1	distal	tubular	well	7	7/12	slight	no
				2	distal	tubular	well	6	6/12	slight	no
30	WT	female	24	1	mid 1	papillary	well	5	5/5	slight	no
				2	mid 1	papillary	well	5	5/5	slight	no
				3	distal	papillary	well	9	9/10	slight	no
31	WT	female	24	1	mid 2	tubular	well	3	3/5	slight	yes
32	WT	female	24	1	mid 2	tubular-papillary	moderate	5	5/5	moderate	yes
				2	mid 2	tubular	well	4	4/5	minimal	no
				3	mid 2	tubular	well	3	3/5	slight	no
				4	mid 2	tubular	well	2	2/5	minimal	no
				5	distal	tubular	moderate	10	10/10	minimal	yes
				6	distal	tubular	moderate	7	7/10	minimal	yes
				7	distal	tubular	moderate	6	6/10	minimal	yes

**Mice**	**Genotype**	**Sex**	**Age (Weeks)**	**Number of Adenocarcinoma**	**Location**	**Morphology**	**Differentiation**	**Number of Slides Seen**	**Relative Number of Slides Seen**	**Associated with Mononuclear Cell Infiltration**	**Associated with Fibrosis**
33	WT	male	24	1	distal	tubular	well	4	4/10	moderate	no
34	WT	female	24	1	mid 2	tubular	well	5	5/5	slight	yes
				2	mid 2	tubular	well	2	2/5	minimal	yes
35	WT	male	24	1	mid 2	tubular-papillary	well	5	5/5	minimal	no
36	WT	female	24	1	mid 2	tubular	well	5	5/5	slight	no
37	WT	male	24	1	distal	tubular	well	3	3/10	slight	no
38	WT	female	24	1	mid 2	tubular	well	4	4/5	/	no
				2	distal	#LEEG!	well	2	2/11	no	no
39	WT	male	24	1	distal	tubular	moderate	10	10/10	moderate	no
40	*Gsdme* KO	female	24	1	mid 2	tubular	well	5	5/5	slight	yes
				2	distal	tubular	well	5	5/15	minimal	no
				3	distal	tubular	well	5	5/15	minimal	no
				4	distal	tubular	well	3	3/15	no	no
41	*Gsdme* KO	male	24	1	mid 2	tubular	well	3	3/5	slight	no
42	*Gsdme* KO	female	24	1	distal	tubular	well	2	2/10	slight	no
43	*Gsdme* KO	female	24	1	mid 2	tubular	moderate	5	5/5	slight	no
				2	distal	tubular	well	6	6/12	minimal	no
				3	distal	tubular	well	3	3/12	minimal	no
44	*Gsdme* KO	female	24	1	mid 2	tubular	well	5	5/5	moderate	no
				2	mid 2	tubular	well	4	4/5	slight	no
				3	mid 2	tubular	well	2	2/5	slight	no
				4	distal	tubular	well	4	4/10	no	no
45	*Gsdme* KO	female	24	1	distal	tubular	well	2	2/10	no	no

Table S5. Overview of the adenocarcinoma characteristics in the Apc[1638N/+] Gsdme KO and Apc[1638N/+] Gsdme WT mice.

Mice	Genotype	Sex	Number of Adenocarcinoma	Location	Associated with Mononuclear Cell Infiltration	Number of Slides Seen	Percentage of Slides Seen	Associated with Fibrosis
1	WT	male	1	proximal	slight	5/5	100	no
2	WT	male	1	proximal	minimal	5/5	100	no
		male	2	proximal	minimal	4/5	80	no
		male	3	mid 2	slight	5/5	100	no
3	WT	male	1	proximal	moderate	5/5	100	no
		male	2	proximal	moderate	4/5	80	no
		male	3	proximal	moderate	3/5	60	no
		male	4	distal	slight	4/5	80	no
4	WT	female	1	mid 2	minimal	2/5	40	no
5	WT	female	1	proximal	moderate	4/5	80	yes
		female	2	proximal	moderate	4/5	80	yes
		female	3	mid 1	moderate	4/5	80	yes
		female	4	mid 1	moderate	4/5	80	yes
		female	5	mid 1	slight	1/5	20	no
6	WT	female	1	mid 1	slight	3/5	60	no
		female	2	mid 2	slight	1/5	20	no
7	WT	male	1	proximal	moderate	4/5	80	yes
		male	2	proximal	moderate	2/5	40	yes
		male	3	mid 1	slight	4/5	80	no
		male	4	mid 1	slight	4/5	80	no
		male	5	mid 1	slight	3/5	60	no
		male	6	mid 1	slight	1/5	20	no
		male	7	mid 2	moderate	2/5	40	no
		male	8	mid 2	slight	2/5	40	no
		male	9	distal	slight	3/5	60	no
8	WT	male	1	proximal	minimal	3/5	60	no
		male	2	distal	minimal	2/5	40	no
9	WT	female	1	proximal	minimal	4/5	80	no
		female	2	mid 1	minimal	2/5	40	no
10	WT	female	1	proximal	slight	2/5	40	no
11	WT	female	1	proximal	slight	1/5	20	no
12	WT	male	1	proximal	slight	5/5	100	no

Mice	Genotype	Sex	Number of Adenocarcinoma	Location	Associated with Mononuclear Cell Infiltration	Number of Slides Seen	Percentage of Slides Seen	Associated with Fibrosis
		male	2	proximal	slight	3/5	60	no
		male	3	mid 1	slight	2/5	40	no
13	WT	male	1	proximal	slight	5/5	100	no
		male	2	proximal	slight	4/5	80	no
		male	3	proximal	slight	3/5	60	no
		male	4	mid 1	minimal	5/5	100	no
14	WT	female	1	proximal	moderate	3/5	60	no
		female	2	mid 1	slight	3/5	60	no
15	WT	female	1	mid 1	slight	4/5	80	no
16	WT	female	1	proximal	slight	4/5	80	no
17	*Gsdme* KO	male	1	proximal	moderate	4/5	80	no
		male	2	mid 1	minimal	5/5	100	no
18	*Gsdme* KO	male	1	proximal	minimal	5/5	100	no
19	*Gsdme* KO	male	1	proximal	moderate	4/5	80	no
		male	2	proximal	moderate	3/5	60	no
		male	3	mid 1	minimal	5/5	100	yes
20	*Gsdme* KO	female	1	proximal	slight	5/5	100	no
		female	2	distal	minimal	3/5	60	no
21	*Gsdme* KO	female	1	mid 1	minimal	3/5	60	no
22	*Gsdme* KO	female	1	proximal	slight	4/5	80	no
		female	2	mid 1	minimal	3/5	60	no
		female	3	distal	minimal	3/5	60	no
23	*Gsdme* KO	female	1	mid 1	slight	2/5	40	no
		female	2	mid 1	slight	2/5	40	no
		female	3	distal	slight	1/5	20	no
24	*Gsdme* KO	female	1	proximal	slight	5/5	100	yes
		female	2	proximal	slight	4/5	80	yes
25	*Gsdme* KO	female	1	proximal	minimal	3/5	60	no
		female	2	proximal	minimal	1/5	20	no
26	*Gsdme* KO	male	1	proximal	slight	3/5	60	no

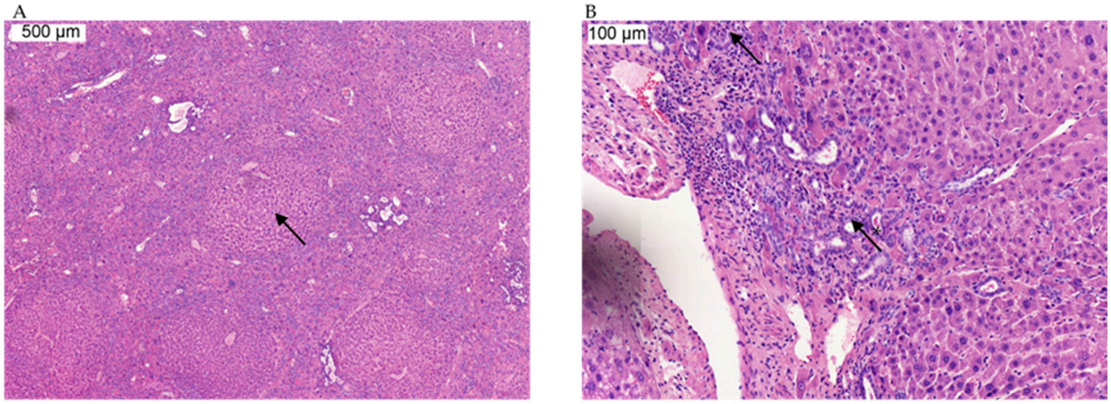

Figure S1. Pathologies in the liver of AOM-treated mice. **A.** Hepatocellular hyperplasia, one example indicated by an arrow. **B.** Liver inflammation: the arrow indicates perilobular mononuclear cell infiltration and the arrow with asterisk indicates biliary duct proliferation. Scale bars are indicated on the images.

Supplementary Information LIVER – CHEMICAL EXPERIMENT

Microscopical analysis of the liver of all 100 mice was performed. Hepatic lesions found in several mice were those of cirrhosis with hypertrophy of hepatocytes; mild portal, pericellular and capsular fibrosis (N = 6/54 WT, N = 7/46 KO); focal areas of dilated sinusoids (N = 17/54 WT, N = 21/46 KO); nodular hepatocellular hyperplasia (N = 7/54 WT, N = 7/46 KO); and biliary duct hyperplasia (N = 13/54 WT, N = 14/46 KO) (Supplementary figure 1). Enlarged liver cells had large nuclei with large nucleoli and intranuclear inclusions (N = 8/54 WT, N = 8/46 KO) (Supplementary figure 1). In literature, these hepatocellular inclusions after AOM treatment have been reported also in rats and would seem to be cytoplasmic invaginations [1]. These 'intranuclear' – intracytoplasmic inclusion bodies are probably proliferations of smooth endoplasmic reticulum (induction of cytochrome P450) induced by AOM. Mice of the C57BL/6 strain show high inducibility of cytochromes in the liver [2].

Dilated sinusoids are associated with hypoxia and can be associated with liver inflammation; or can be seen around hepatic nodules, due to hemodynamic changes during sacrifice. Foci of hyperplasia were sometimes demarcated by fibrous tissue that accentuated the nodules (macroscopically visible; Supplementary figure 1). Hepatic cells in these nodules were usually of normal size or larger or smaller than normal hepatocytes. These lesions were also reported in literature in rats [1]. Cirrhosis develops as a chronic stage after acute liver injury (necrosis) and is seen after administration of several xenobiotics [3]. The cirrhosis encountered here is most probably related to the AOM treatment.

In one WT mouse a focal nodular lesion was seen. In a focal area of biliary duct proliferation there was deposition of crystalloid and strongly basophilic substances, most probably calcium, in the lumina of the ducts associated with degeneration of the biliary walls. In one *Gsdme* KO mouse a nodular adenoma was found in the liver. In addition, there were some other lesions seen: congestion (N = 40/54 WT, N = 40/46 KO), which is probably related to circulation disorders during sacrifice; hepatocellular macro- and microvesicular vacuolation (N = 24/54 WT, N = 24/46 KO) which is a result of fat accumulation and is related to the nutritional status of the animal, but can also be related to hepatic dysfunction (fatty change). Moreover, microgranulomes (N = 41/54 WT, N = 39/46 KO), small aggregates of inflammatory cells grouped around small zones of degenerate hepatocytes, were found. Finally, portal inflammation of mononuclear cells (N = 6/54 WT, N = 11/46 KO) was observed, which can be associated with biliary tract lesions. Lastly, in one *Gsdme* KO mouse extramedullar hematopoiesis was seen. Hematopoiesis can occur in the liver when normal hematopoiesis in the bone marrow and/or spleen is disturbed, for example when the bone marrow and/or spleen are

invaded with tumor cells (lymphoma). For none of those hepatic lesions we were able to find a statistical significant difference between WT and *Gsdme* KO mice.

Supplementary Information LUNGS - CHEMICAL EXPERIMENT

Lungs from 22 *Gsdme* KO and 16 WT mice were analyzed. These lungs revealed red patches at necropsy. Histological examination revealed congestion (N = 12/16 WT, N = 12/22 KO), edema (N = 1/16 WT, N = 2/22 KO) and/or areas of atelectasis (N = 4/16 WT, N = 6/22 KO). These lesions are probably related to hemodynamic changes during sacrifice. Two WT and two *Gsdme* KO mice showed hemorrhage of the lungs. One *Gsdme* KO mouse showed focal, minimal, interstitial mononuclear cell infiltration. In one WT mouse pneumonia was seen. This animal presented at necropsy with purple-colored patches on the lung surfaces. Microscopically, a marked inflammatory infiltrate of neutrophilic granulocytes and mononuclear cells was multifocally present in the lung alveoli and septa. This inflammation can have different causes. In aged mice it has been associated with aspiration of foreign material in relation to general bad health.

Supplementary Information LIVER and LUNGS – GENETIC EXPERIMENT

In the liver, hyperemia (N = 18/24 WT, N = 10/13 KO) and dilated sinusoids (N = 7/24 WT, N = 6/13 KO) were observed, which were probably related to circulation disorders during sacrifice. Microgranulomes were also found (N = 15/24 WT, N = 7/13 KO). This lesion was considered to be background pathology. In one *Apc*$^{1638N/+}$ *Gsdme* KO animal the severity was marked. None of these lesions was significantly different between *Apc*$^{1638N/+}$ *Gsdme* WT and *Apc*$^{1638N/+}$ *Gsdme* KO mice.

In the lungs, hyperemia (N = 12/24 WT, N = 8/13 KO) and hemorrhages (N = 3/24 WT, N = 3/13 KO) were found, which were probably related to circulation disorders during sacrifice. In one *Apc*$^{1638N/+}$ *Gsdme* WT and one *Apc*$^{1638N/+}$ *Gsdme* KO mouse lymphocytic interstitial infiltration was found which indicated (focal) inflammation in the lungs. In one *Apc*$^{1638N/+}$ *Gsdme* WT mouse areas of atelectasis and emphysema were observed, which were probably related to respiration disorders during sacrifice. None of these lesions were significantly different between *Apc*$^{1638N/+}$ *Gsdme* WT and *Apc*$^{1638N/+}$ *Gsdme* KO mice.

Genotyping protocol

Gsdme

Table S6. Primers for Gsdme genotyping.

Genotype	Forward Primer	Reverse Primer	Amplicon size (bp)
**Floxed (PCR 1)**	GAGATGGCGCAACGCAATTAATG	CACCCCAGGATCAGCTTGGATATGG	343
**Pre Cre (PCR 2)**	GGGATCTCATGCTGGAGTTCTTCG	AGTAGAAGGGTGGCAGACATCATGG	558
**Wildtype (PCR 3)**	CTTTGTCCACTTCAGGGTTGCTTCC	AGTAGAAGGGTGGCAGACATCATGG	561

Table S7. Mastermix for the Gsdme genotyping PCR.

Reagents	Volume (µl)
Water	19.0
5x Green GoTaq reaction buffer (Promega)	5.0
Forward primer (100µM, IDT)	0.25
Reverse primer (100µM, IDT)	0.25
dNTP (10mM, Promega)	0.25
GoTaq DNA Polymerase (5U/µl, Promega)	0.1
DNA	1.0

Table S8. Cycling parameters Gsdme genotyping PCR.

Temperature (°C)	Time	
94	5 min	
94	45 sec	
63	45 sec	35×
72	45 sec	
72	7 min	
4	5 min	

Apc^{1638N}

Table S9. Primers for Apc^{1638N} genotyping.

Genotype	Forward Primer	Reverse Primer	Amplicon size (bp)
**Wildtype**	TCAGCCATGCCAACAAAGTCA	GGAAAAGTTTATAGGTGTCCCTTCT	216
Apc^{1638N}	TCAGCCATGCCAACAAAGTCA	GCCAGCTCATTCCTCCACTC	~400

Table S10. Mastermix for the Apc^{1638N} genotyping PCR.

Reagents	Volume (µl)
Water	19.0
5x Green GoTaq reaction buffer (Promega)	5.0
Forward primer (33µM, IDT)	0.20
Reverse primer 1 (33µM, IDT)	0.20
Reverse primer 2 (33µM, IDT)	0.20
dNTP (10mM, Promega)	0.25
GoTaq DNA Polymerase (5U/µl, Promega)	0.1
DNA	1.0

Table S11. Cycling parameters Apc^{1638N} genotyping PCR.

Temperature (°C)	Time	
94	5 min	
94	45 sec	
53	45 sec	35×
72	45 sec	
72	7 min	
4	5 min	

References

1. Ward, J. M. Dose response to a single injection of azoxymethane in rats. Induction of tumors in the gastrointestinal tract, auditory sebaceous glands, kidney, liver and preputial gland. *Vet. Pathol.* **1975**, *12*, 165–177.
2. Nolte, T.; Brander-Weber, P.; Dangler, C.; Deschl, U.; Elwell, M. R.; Greaves, P.; Hailey, R.; Leach, M. W.; Pandiri, A. R.; Rogers, A.; et al. Nonproliferative and Proliferative Lesions of the Gastrointestinal Tract, Pancreas and Salivary Glands of the Rat and Mouse. *J. Toxicol. Pathol.* **2016**, *29*, 1S–125S.
3. Greaves, P. Liver and pancreas, In *Histopathology of Preclinical Toxicity Studies*, Elsevier: Amsterdam, The Netherlands, 2007; ISBN: 9780444538611

© 2019 by the authors. Licensee MDPI, Basel, Switzerland. This article is an open access article distributed under the terms and conditions of the Creative Commons Attribution (CC BY) license (http://creativecommons.org/licenses/by/4.0/).
